# Initiation of antidepressant medication in people with type 2 diabetes living in the United Kingdom—A retrospective cohort study

**DOI:** 10.1002/pds.5484

**Published:** 2022-06-10

**Authors:** Kingshuk Pal, Manuj Sharma, Naaheed M. Mukadam, Irene Petersen

**Affiliations:** ^1^ Department of Primary Care and Population Health UCL London UK; ^2^ Division of Psychiatry Faculty of Brain Sciences, UCL London UK

**Keywords:** antidepressant, depression, type 2 diabetes

## Abstract

**Introduction:**

Depression is a common comorbidity in people with type 2 diabetes and it is associated with poorer outcomes. There is limited data on the treatments used for depression in this population. The aim of this study was to explore the rates of initiation of antidepressant prescriptions in people with type 2 diabetes in the UK and identify those most at risk of needing such treatment.

**Research Design and Methods:**

This was a retrospective cohort study using data from IQVIA Medical Research Data (IMRD)‐UK data. Data from general practices in IMRD‐UK between January 2008 and December 2017 were used for this study.

**Results:**

The overall rates of antidepressant prescribing were stable over the study period. The rate of initiation of antidepressant medication in people with type 2 diabetes was 22.93 per 1000 person years at risk (PYAR) with a 95%CI 22.48 to 23.39 compared to 16.89 per 1000 PYAR (95%CI 16.77 to 17.01) in an age and gender matched cohort. The risk of being prescribed antidepressant medication with age had a U‐shaped distribution with the lowest risk in the 65–69 age group. The peak age for antidepressant initiation in men and women was 40–44, with a rate in men of 32.78 per 1000 PYAR (95% CI 29.57 to 36.34) and a rate in women of 46.80 per 1000 PYAR (95% CI 41.90 to 52.26). People with type 2 diabetes with in the least deprived quintile had an initiation rate of 19.66 per 1000 PYAR (95%CI 18.67 to 20.70) compared to 27.19 per 1000 PYAR (95%CI 25.50 to 28.93) in the most deprived quintile, with a 32% increase in the risk of starting antidepressant medication (95%CI 1.22 to 1.43).

**Conclusions:**

People with type 2 diabetes were 30% more likely to be started on antidepressant medication than people without type 2 diabetes. Women with type 2 diabetes were 35% more likely than men to be prescribed antidepressants and the risks increased with deprivation and in younger or older adults, with the lowest rates in the 65–69 year age band. The rates of antidepressant prescribing were broadly stable over the 10‐year period in this study. The antidepressant medications prescribed changed slightly over time with sertraline becoming more widely used and fewer prescriptions of citalopram.

**Keypoints**

- The highest risk groups for being prescribed antidepressant medication in people with type 2 diabetes the UK were women, middle aged or older patients and more deprived populations.
- Sertraline is now the most commonly initiated antidepressant in people with type 2 diabetes in the UK.

**Plain language summary**

Depression is a common problem in people with type 2 diabetes and it is associated with poorer health outcomes. The aim of this study was to explore the rates of starting antidepressant medicines in people with type 2 diabetes in the United Kingdom and identify which patients with type 2 diabetes were most likely to be started on antidepressant medication. This study looked at historical data stored in electronic medical records in the IQVIA Medical Research Data (IMRD)‐UK data between January 2008 and December 2017. People with type 2 diabetes were 30% more likely to be started on antidepressant medication than people without type 2 diabetes. Women with type 2 diabetes were 35% more likely than men to be prescribed antidepressants and the risks increased with deprivation and in younger or older adults, with the lowest rates in the 65–69 year age band. The rates of antidepressant prescribing were broadly stable over the 10‐year period in this study. The antidepressant medication that people with type 2 diabetes were started on changed over time, with sertraline becoming more widely used and fewer prescriptions of citalopram.

## 1 INTRODUCTION

According to primary care registers, diabetes is the third most common long‐term condition in England after hypertension and depression, and it affects around 6% of the population. Diabetes causes significant morbidity and mortality: it can shorten life expectancy by 8–10 years if diabetes is poorly controlled.^1^ Around four million deaths worldwide are related to diabetes and direct healthcare costs range from 2.5% to 15% of annual heathcare budgets—around the world over 500 billion US dollars is spent on treating diabetes, with the majority of costs spent on treating diabetes related complications.^2^, ^3^ The presence of depression increases the risk of poorer outcomes in diabetes as it is associated with poor glycemic control and increased rates of complications.^4^, ^5^ Depression has been found to significantly increase the likelihood of being diagnosed with diabetes.^6^, ^7^

There are a number of studies that have examined this bidirectional relationship between diabetes and depression. The relationship between the two might be partly due to shared underlying pathophysiology driven by changes in hormones in the hypothalamus–pituitary–adrenal cortex axis and sympathetic nervous system.^8^, ^9^ Both conditions are also associated with subclinical inflammation.^10^ There are also behavioural factors and complications associated with these conditions that link them with poorer self‐care requiring healthier lifestyles and medication adherence.^11^ The net result is a shared increase in vulnerability to these common chronic conditions and poorer outcomes (including increased mortality) where they co‐exist.^12^

Estimates of the prevalence of depression in people with type 2 diabetes vary widely, ranging from 0% to 39% in a systematic review.^13^ However, there are not many studies on populations within the UK. A retrospective cohort study following 3225 people in Hertfordshire, UK estimated the prevalence of depression in people with diabetes as 8.5%.^14^ Risk factors for developing depression include the presence of microvascular and macrovascular complications, female gender, being single, external locus of control and previous depression.^15^, ^16^ The prevalence of depression in the general population in UK has been estimated to be 3.3%.^17^ When examining primary care records, depression recorded by general practitioners (GPs) has lower incidence rates that epidemiological studies as GPs increasingly use symptoms rather than labels to code the illness and the incidence rate from studies on electronic records has been estimated at 2.5%.^18^ Looking at rates of antidepressant prescriptions avoids such risks of coding issues for common mental health problems and can help identify patients most at risk of needing to start antidepressant medication and likely to be higher risk of developing diabetes‐related complications.

The aim of this study was to explore the rates of initiation of antidepressant prescriptions in people with type 2 diabetes in the United Kingdom and identify those most at risk of needing such treatment.

## 2 RESEARCH DESIGN AND METHODS

This was a retrospective cohort study using data from IQVIA Medical Research Data (IMRD)‐UK data. The IMRD‐UK database contains electronic primary care health records for approximately 12 million patients in the UK. The majority of diabetes and depression is usually treated and managed in primary care hence diagnoses, monitoring and treatments should be captured by IMRD‐UK. Multiple validation studies have shown IMRD‐UK data to be generalizable to the wider UK population.^19^, ^20^ Data quality was ensured by using practices which had reached the standard for acceptable mortality rate (AMR) and acceptable computer usage (ACU). Acceptable mortality rates were reached when death rates recorded in the electronic database matched expected standardised mortality ratios and acceptable computer usage was reached when records included at least one medical record, additional health data and two therapy records per patient per year. The combination of AMR and ACU can be used as a marker of data quality.^21^, ^22^ Data from general practices in IMRD‐UK between 1 January 2008 and 31 December 2017 were used for this study.

Definitions for key variables and inclusion criteria are described below:

Eligible participants were people diagnosed with type 2 diabetes aged 35–99 years and who were permanently registered. People who had been registered for less than 9 months at the practice prior to first treatment were excluded for incidence calculations (but not prevalence calculations) as these cases were more likely to represent delayed coding of patients with pre‐existing diabetes. Patients with diabetes diagnosed <35 and on insulin were excluded as they were likely to have type 1 diabetes.

Patients with diabetes were defined as people using a previously validated algorithm with at least two of the following records^1^: a diagnostic code for diabetes,^2^ supporting evidence of diabetes, for example, screening for diabetic retinopathy or^3^ treatment for diabetes.^23^ The codes used to identify people living with type 2 diabetes can be found in Appendix S1.

A cohort of patients from the same practice, matched for age and gender, and without type 2 diabetes were identified for the comparison group. A sample size of up to five times the population with diabetes was used.^24^ The matching was done in 5 year bands between 35 and 99.

Prescriptions for these therapies were identified using the drug code list based on Section 4.3 of the British National Formulary December 2017 update and used generic names of all SSRIs, TCAs, Venlafaxine and Mirtazapine.^25^ Low doses of tricyclic antidepressants and prescriptions for duloxetine were excluded as they may be used to treat diabetic neuropathy. Prevalent use of antidepressant medication in a calendar year was calculated as the proportion of patients registered with a practice for the whole of that year who were issued a prescription for antidepressant medication.

The incidence rate for starting patients on antidepressant medication was calculated for each calendar year as the rate of new antidepressant prescriptions per 100 person‐years at risk (PYAR). This was calculated as the total number of people given their first ever prescription of an antidepressant medication after diagnosis with type 2 diabetes in that year divided by the total person–years at risk. Time at risk was calculated as the time from entry into the study until the first prescription of an antidepressant medication. Participants with any previous prescriptions for antidepressant medication prior to a diagnosis of type 2 diabetes were excluded from this calculation. The study population for this calculation was an incident cohort of people with type 2 diabetes who developed type 2 diabetes 9 months or longer after joining the practice with no history of antidepressant use prior to the diagnosis of type 2 diabetes. The comparison cohort was an age and gender matched cohort from the same practice. The rate of new antidepressant prescribing was plotted over time for all and individual antidepressant medication.

For descriptive purposes, we also estimated the prevalence of antidepressant medication use in people with type 2 diabetes for each calendar year. This was calculated as the number of individuals with type 2 diabetes prescribed antidepressant medication in a year, divided by the total number of people in that year who had a diagnosis of type 2 diabetes. Only people who were registered with the practice for the whole year were included in the calculation.

### 2.1 Statistical analysis

To analyse the incidence data, we used a Poisson regression to examine the relationship between initiation of an antidepressant medication and gender, age and Townsend quintile. Townsend scores were used as a measure of social deprivation, where social deprivation was assigned quintiles with 1 being the least deprived and 5 being the most deprived.^26^

Models were estimated separately for men and women and included a random effect to account for clustering by practice. We calculated the ratio of and difference between incidence rates of new prescriptions of antidepressant use in people with and without type 2 diabetes with 95% confidence intervals. To examine the impact of duration of diabetes on the risk of starting on antidepressant medication, we looked at the time to first prescription from diagnosis of type 2 diabetes in the incident cohorts of patients newly diagnosed with type 2 diabetes during the study period and compared this with duration from a randomly generated index date (pseudo‐diagnosis date) for the matched comparison cohort. We plotted a Kaplan–Meier curve of time to first prescription of antidepressant stratified by gender, age and Townsend score.

We used Poisson regression to examine the relationship between antidepressant initiation and gender, age, and Townsend score. There was a significant interaction between age and gender, so the results for age were stratified by sex. Models included a random effect to account for clustering by practice.

Analyses were conducted with Stata software version 16.0 (Stata Corporation, USA).

### 2.2 Ethics

The data provider (IQVIA) obtained overall ethical approval for the use of the data in scientific research from the South East Medical Research Ethics Committee (MREC/03/01/073). Approval to undertake this study was obtained from Scientific Review Committee in 2018 (SRC Reference Number: 18THIN032).

## 3 RESULTS

**FIGURE 1:**
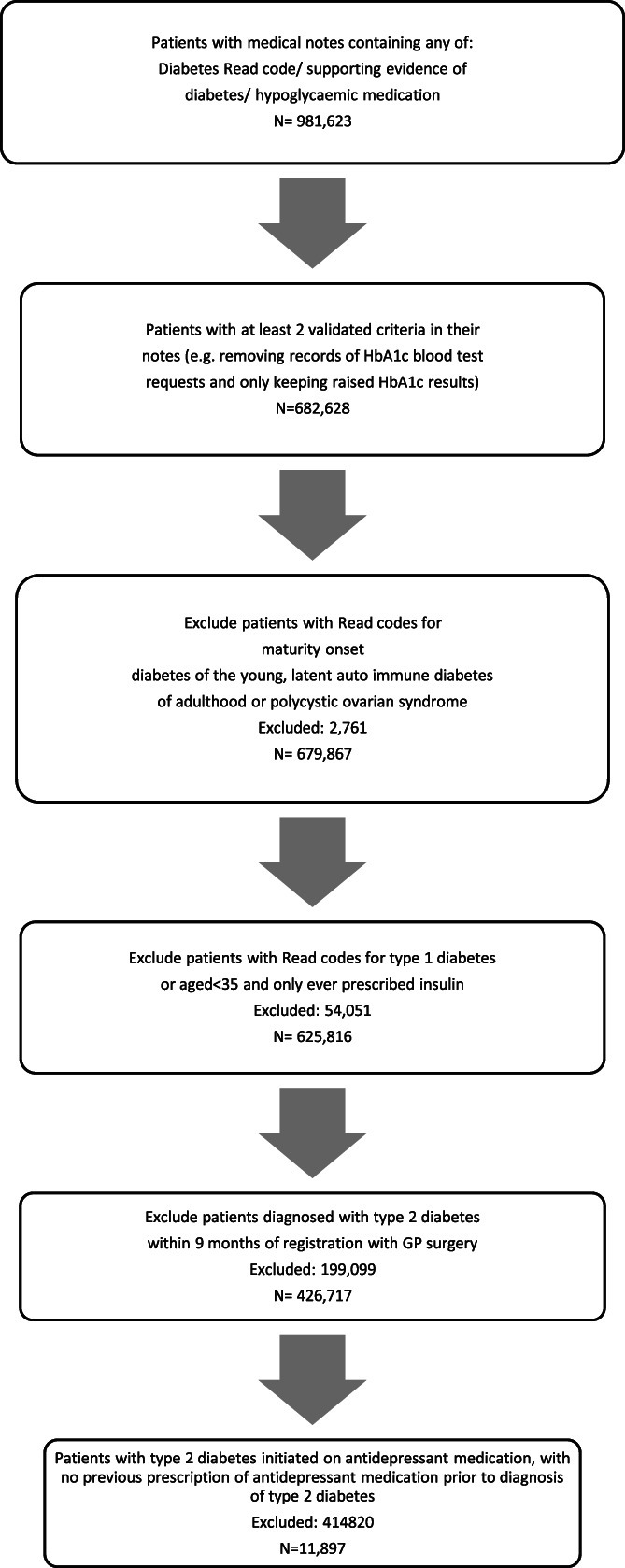
Flowchart showing participants included in study

There were 625 816 people who fit the criteria for a diagnosis of type 2 diabetes in the cohort, of which 426 717 were new cases diagnosed between 1 January 2008 and 31 December 2017, with a total follow‐up time of 7 658 463 years. Demographic details about the cohort have been published previously,^27^ but a brief summary is provided in the following sections. About 11 897 suitable patients with type 2 diabetes and no previous exposure to antidepressant medication were started on an antidepressant after their diagnosis of type 2 diabetes (Figure 1). About 48.8% of participants were female (*N* = 6097), with a mean age of 58 years at diagnosis for type 2 diabetes. The mean age of diagnosis of type 2 diabetes for men was 59 (*N* = 5800). When looking at initiation of antidepressant medication in people with type 2 diabetes and the comparison group, there were significant interactions between gender and Townsend score (deprivation index) so results have been stratified by gender and Townsend score. The period prevalence of antidepressant prescribing was relatively stable over time in both the type 2 diabetes and comparison cohort. The prevalence in 2018 was 31.04 per 1000 individuals with type 2 diabetes and 27.36 per 1000 individuals in the age and gender matched cohort (Supporting Information, Figure S1).

In men with type 2 diabetes, the initial peak for starting on antidepressants was between 40 and 44 (with a rate of 32.78 per 1000 PYAR, 95% CI 29.57 to 36.34) (Table 1). The rate of prescribing then dropped to 13.70 per 1000 PYAR (95% CI 12.60 to 14.88) in the 65–69 age band, before steadily increasing to 34.18 per 1000 PYAR (95% CI 30.64–38.12) in men over 85. In women with type 2 diabetes, the peak for starting on antidepressants was between 40 and 44 (with a rate of 46.80 per 1000 PYAR, 95% CI 41.90–52.26). The rate of prescribing then dropped to 20.73 per 1000 PYAR (95% CI 18.97–22.65) in the 65–69 age band, before steadily increasing to 31.43 per 1000 PYAR (95% CI 28.47–34.70) in women over 85.

**TABLE 1:** Incidence rates and Incidence rate ratios of starting antidepressant medication by age band in people with type 2 diabetes

Age band	Rate per 1000 PYAR	(95% CI)		IRR	(95% CI)
Men with type 2 diabetes					
35–39	28.42	(24.32–33.22)		1.98	(1.57–2.48)
40–44	32.78	(29.57–36.34)		2.29	(2.00–2.81)
45–49	25.20	(23.05–27.56)		1.67	(1.46–2.00)
50–54	22.85	(21.13–24.72)		1.64	(1.45–1.93)
55–59	20.00	(18.52–21.61)		1.47	(1.28–1.70)
60–64	15.95	(14.72–17.28)		1.16	(1.00–1.33)
65–69	13.70	(12.60–14.88)		1	
70–74	15.33	(14.06–16.70)		1.14	(0.99–1.31)
75–79	18.72	(17.12–20.47)		1.43	(1.25–1.67)
80–84	24.16	(21.84–26.73)		1.83	(1.59–2.15)
85+	34.18	(30.64–38.12)		2.50	(2.27–3.11)
Women with type 2 diabetes					
35–39	40.05	(34.92–45.93)		1.84	(1.51–2.24)
40–44	46.80	(41.90–52.26)		2.32	(1.96–2.75)
45–49	38.92	(35.20–43.03)		1.85	(1.57–2.18)
50–54	35.19	(32.16–38.51)		1.80	(1.55–2.09)
55–59	27.90	(25.45–30.59)		1.29	(1.10–1.51)
60–64	20.21	(18.81–22.81)		1.04	(0.89–1.21)
65–69	20.73	(18.97–22.65)		1	
70–74	21.06	(19.27–23.03)		1.04	(0.90–1.21)
75–79	25.06	(23.02–27.28)		1.22	(1.05–1.41)
80–84	31.19	(28.54–34.10)		1.50	(1.30–1.74)
85+	31.43	(28.47–34.70)		1.56	(1.34–1.81)

Abbreviations: CI, Confidence interval; IRR, Incidence rate ratio; PYAR, person years at risk.

**FIGURE 2:**
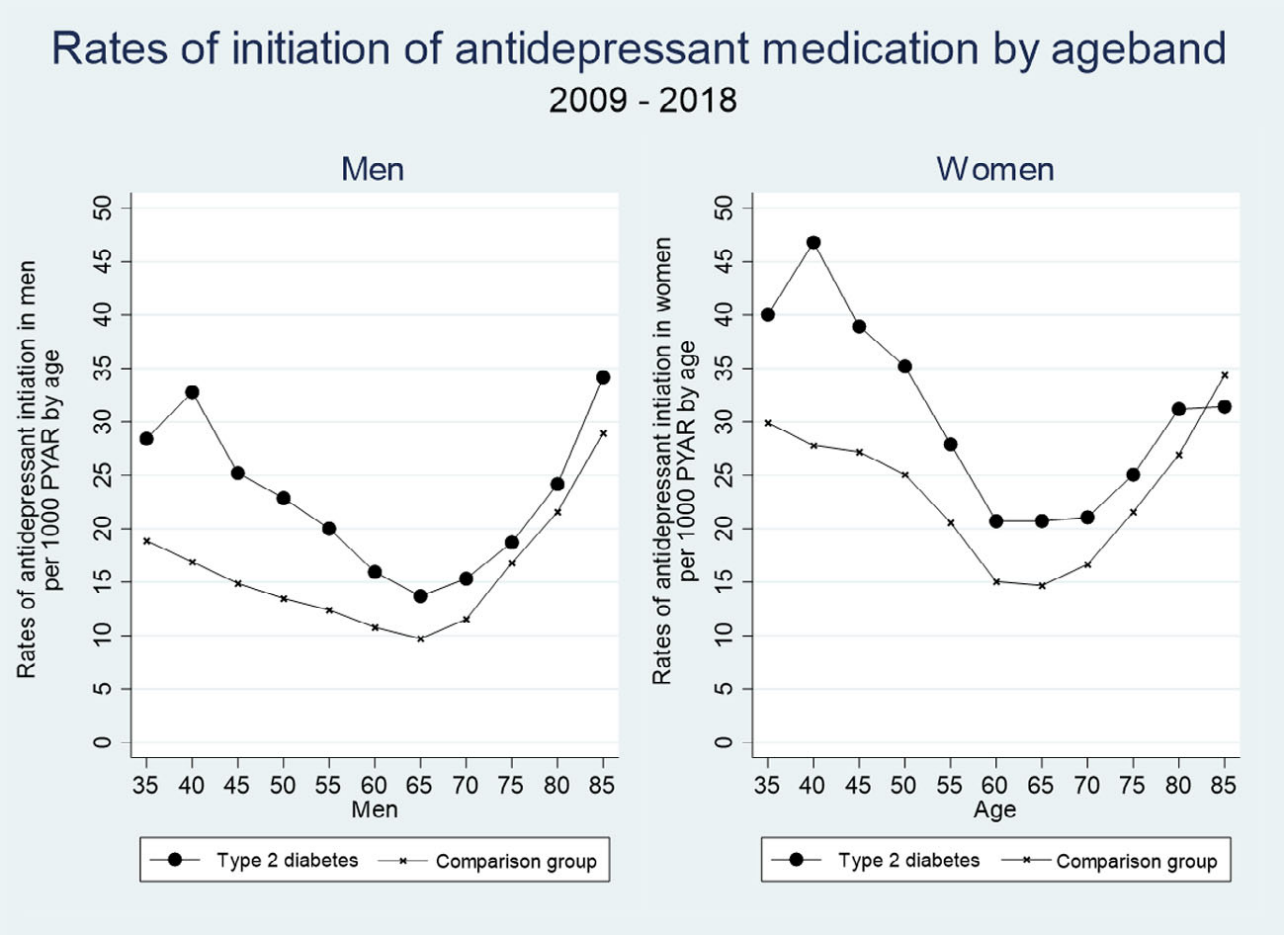
Rates of initiation of antidepressant medication by age band in men and women

The risk of being prescribed an antidepressant medication appeared to have a U‐shaped distribution with the lowest risk in the 65–69 age group (Figure 2). The main difference in the rates of antidepressant medication initiation between people with type 2 diabetes and the comparison group was in younger people, peaking in between the age of 40–45, with the differences reducing with increasing age (Figure 2).

People with type 2 diabetes were 1.29 (95%CI 1.26–1.33) times more likely to be started on antidepressant medication than people in an age and gender matched cohort of people from the same practices (Table 1). About 11.1% (95%CI 10.9–11.4) of individuals with type 2 diabetes were initiated on antidepressant medication within the first 5 years of diagnosis, compared to 9% (95% CI 8.9–9.1) of the age and gender matched comparison group. A Kaplan–Meier plot of time to first prescription of antidepressant medication in people with type 2 diabetes and the comparison group can be found in Supporting Information, Figure S2.

A summary table of risk of starting on antidepressant medication and socio‐demographic characteristics can be found in Table 2. The risk of a woman with type 2 diabetes being prescribed an antidepressant was 1.35 times that of men (95% CI 1.29–1.43). People with type 2 diabetes with in the least deprived quintile had an initiation rate of 19.66 per 1000PYAR (95%CI 18.67–20.70) compared to 27.19 per 1000PYAR (95%CI 25.50–28.93) in the most deprived quintile, with a 32% increase in the risk of starting antidepressant medication (95%CI 1.22–1.43) in participants in the most deprived quintile. There was no significant change in rates of starting on antidepressant medication over time.

**TABLE 2:** Incidence rates and Incidence rate ratios of starting antidepressant medication by gender, deprivation, and calendar year

	Rate per 1000 PYAR	(95% CI)	IRR	(95% CI)
*Overall*				
Comparison group	16.89	(16.77–17.01)	1	
Type 2 diabetes	22.93	(22.48–23.39)	1.29	(1.26–1.33)
*People with type 2 diabetes*				
Men	19.87	(20.01–21.20)	1	
Women	27.67	(26.88–28.47)	1.35	(1.29–1.43)
*Townsend score*				
(Least deprived) 1	19.66	(18.67–20.70)	1	
2	20.56	(19.52–21.66)	1.04	(0.97–1.12)
3	23.26	(22.12–24.47)	1.15	(1.07–1.24)
4	25.13	(23.84–26.50)	1.23	(1.14–1.33)
(Most deprived) 5	27.19	(25.50–28.93)	1.32	(1.22–1.43)
*Calendar year*				
2008	20.70	(17.58–24.37)	1	
2009	24.53	(22.45–26.80)	1.12	(0.90–1.41)
2010	24.92	(23.24–26.71)	1.08	(0.88–1.34)
2011	25.42	(23.95–26.99)	1.12	(0.91–1.38)
2012	25.50	(24.15–26.92)	1.17	(0.96–1.44)
2013	20.74	(19.61–21.95)	0.94	(0.77–1.16)
2014	22.65	(21.48–23.88)	1.03	(0.84–1.26)
2015	22.00	(20.81–23.26)	1.04	(0.85–1.28)
2016	21.52	(20.27–22.83)	0.98	(0.79–1.20)
2017	21.92	(20.61–23.30)	1.02	(0.82–1.25)

Abbreviations: CI, Confidence interval; IRR, Incidence rate ratio; PYAR, person years at risk.

**FIGURE 3:**
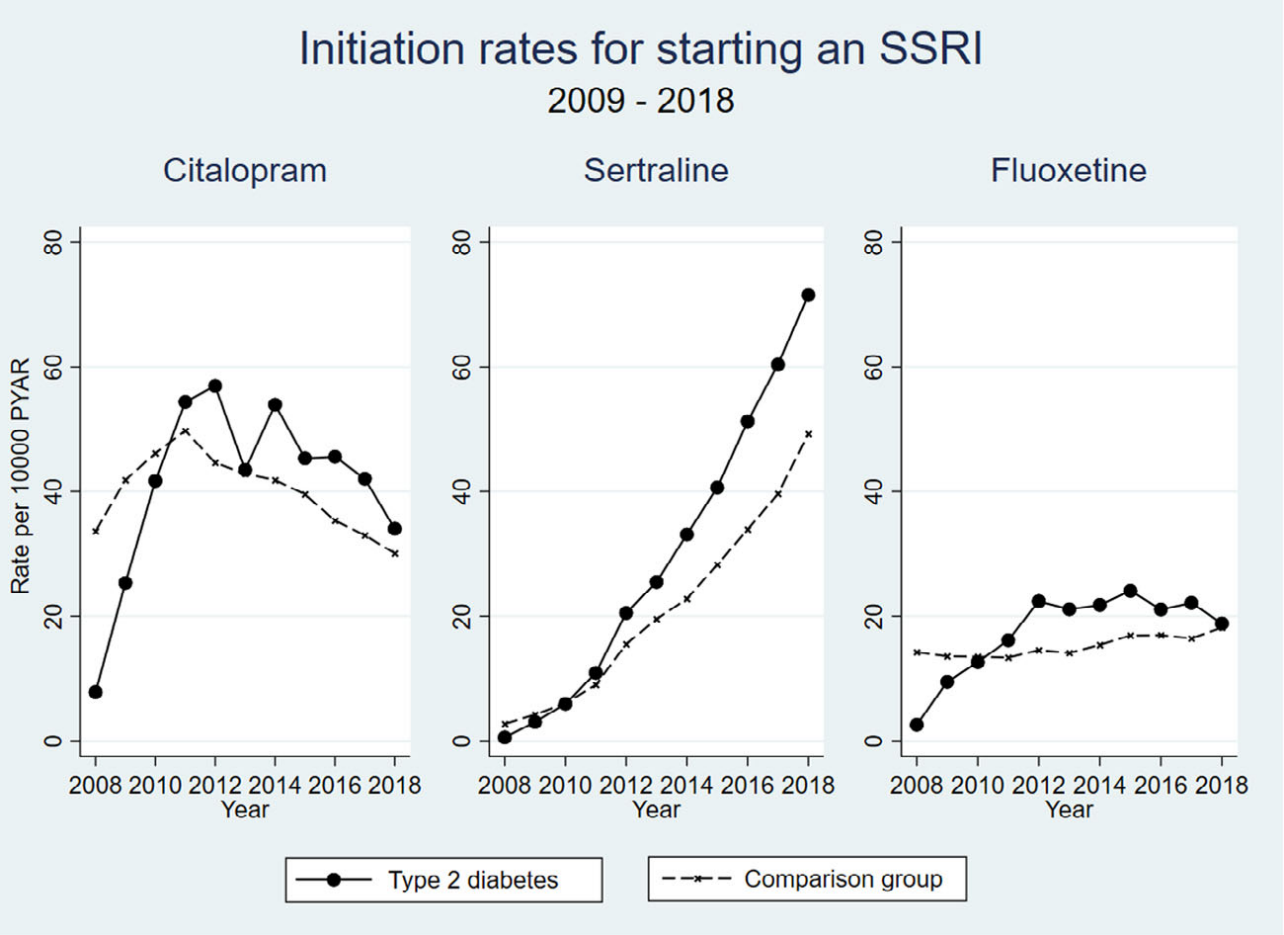
Comparison of initiation rates for starting citalopram, sertraline, and fluoxetine over time

The most commonly used antidepressants started in people with type 2 diabetes were SSRIs—with Citalopram and Sertraline being most widely used (Figure 3). Citalopram prescribing has been decreasing since 2011 while Sertraline prescriptions have been rising since 2009. Initiation of Fluoxetine increased from 2008 to 2012 and has since remained relatively stable. Trends in the comparison group were similar.

## 4 CONCLUSIONS

### 4.1 Main findings

People with type 2 diabetes were 30% more likely to be started on antidepressant medication than people without type 2 diabetes. Women with type 2 diabetes were 35% more likely than men to be prescribed antidepressants and the risks increased with deprivation and in younger or older adults, with the lowest rates in the 65–69 year age band. The rates of antidepressant prescribing were broadly stable over the 10‐year period in this study. The antidepressant medications prescribed changed slightly over time with sertraline becoming more widely used and fewer prescriptions of citalopram.

### 4.2 Comparability with other findings

A recent systematic review of antidepressant use in type 2 diabetes highlighted a dearth of studies on the use of antidepressant medication in people with type 2 diabetes, with only seven included studies, of which six were in the USA and one in Australia.^28^ The prevalence of antidepressant use in the populations studied in the USA varied from 18% to 87% and in Australia it was 37%. Data from other countries is mostly available on the incidence of diagnosed depression in patients with type 2 diabetes. Such studies in people with type 2 diabetes in other countries have suggested lower prevalence of diagnosed depression e.g. Quebec^29^—9.47/1000 PYAR, Saskatchewan^30^—6.5/1000 PYAR and Taiwan^31^—7.03/1000 PYAR. The initiation rate of antidepressant prescriptions use (23.7/1000 PYAR) was higher in this study, but this may reflect the fact that antidepressant medication can be used for other common mental health problems like anxiety, and the presence of higher rates of antidepressant medication prescribing in the United Kingdom.^32^, ^33^ The risk factors identified for depression in people with type 2 diabetes by Lunghi et al.^29^ were similar to this study. They found the risk of incident depression was associated with age, gender and socioeconomic status, with a U‐shaped curve for incidence against age. A cohort study of antidepressant prescribing in the general population across Europe showed similar trends with a dip in rates of prescriptions in the seventh decade before rising again later in life.^32^ Brown et al.^30^ did not find an association between diabetes and depression, however the comparison cohort was not matched by age and the mean age of the comparison cohort was significantly younger (and would have a higher risk of developing depression). Chen et al.^31^ found the highest prevalence of depression in younger people but grouped together all patients over 65 so trends in older people were not described.

Trends in antidepressant medication used in people with type 2 diabetes are similar to trends in antidepressant prescribing in the general population, with sertraline becoming increasingly popular and citalopram less popular.^33^

### 4.3 Results by stratification

There is mixed evidence in the general population about the changes in prevalence of depression in older adults.^34^ Reasons for increased risk of depression in older age could include physical disability, higher cognitive impairment, relative proportion of women and lower socioeconomic status.^35^ The evidence from this study and Lunghi et al suggests that, at least in people with type 2 diabetes, the incidence of depression increases in later life. The slight decrease in incidence around 65 may indicate a reduction in life stress around the time of retirement. In nondiabetic populations, the incidence of depression may be higher in older women^36^ while the added burden and complications of living with type 2 diabetes may have more of an impact on men.

### 4.4 Strengths and weaknesses

This study has a number of strengths. The data were drawn from a large cohort of people with type 2 diabetes, followed up over 10 years, with detailed information about their diabetes care and prescriptions. Most people with T2DM and depression in the United Kingdom will receive their care, treatment and prescriptions within general practice and will have been well recorded in IMRD‐UK and IMRD‐UK data has also been shown to representative of the UK population.

This study also has some limitations that need to be borne in mind when considering the results. A very small minority of patients will receive care for these conditions away from general practices or receive care outside of NHS settings which might not be captured in IMRD‐UK. These small numbers are unlikely to substantially affect our results. We have excluded low dose TCA and those prescribed duloxetine from our analysis as these are more likely to be used for neuropathic pain. There will also be differences between rates of diagnosed depression and rates of antidepressant medication prescribing. Not all depression will be treated by medication and many cases will be treated with psychological therapy in combination with or instead of medical therapy. Antidepressant medication can also be used to treat other conditions like anxiety, obsessive–compulsive disorder, and panic attacks—therefore, antidepressant prescription records will most closely correlate with moderate to severe episodes of common mental health problems.

While the literature suggests that outcomes are poorer for people with type 2 diabetes with co‐morbid depression, what is less clear is whether treating depression has reversed that effect. Further studies examining the impact on glycaemic control of treating depression and comparing the effects of different antidepressants would be beneficial in exploring the best treatment modality for co‐morbid type 2 diabetes and depression.

## 5 CONCLUSION

People with type 2 diabetes were 30% more likely to be started on antidepressant medication than people without type 2 diabetes. Women with diabetes were 35% more likely to prescribed antidepressants than men and the risks increased with deprivation, extremes of age and treatment with insulin. The rates of antidepressant prescribing were broadly stable over the 10‐year period in this study. The antidepressant medications prescribed changed slightly over time with Sertraline becoming more widely used and fewer prescriptions of Citalopram.

## AUTHOR CONTRIBUTIONS

Kingshuk Pal and Irene Petersen conceived and designed the study. Irene Petersen supervised the research. Kingshuk Pal acquired the data. Kingshuk Pal, MS, NM and Irene Petersen analysed and interpreted the data. Kingshuk Pal wrote the first draft and all authors revised the manuscript. The corresponding author attests that all listed authors meet authorship criteria and that no others meeting the criteria have been omitted. The lead author (KP) takes responsibility for the content of the article and confirms that this manuscript is an honest, accurate, and transparent account of the study being reported.

## CONFLICT OF INTEREST

The authors declare no conflict of interest.

## FUNDING INFOMRATION

Kingshuk Pal is funded by the National Institute for Health Research (NIHR) School for Primary Care Research. The views expressed are those of the author(s) and not necessarily those of the NIHR or the Department of Health and Social Care. Naaheed M. Mukadam was funded by an Alzheimer's Society Senior Research Fellowship (SF‐18b‐001) and is supported by UCLH NIHR Biomedical Research Centre.

## Supporting information


**Supplementary Figure S1** Period prevalence of antidepressant medication prescriptions in people diagnosed with type 2 diabetes
**Supplementary Figure S2**: Time to first prescription of antidepressant medication in people with type 2 diabetes and the comparison group

**Appendix S1** Supporting Information
