# Supplementary material for: Initiation of antidepressant medication in people with type 2 diabetes living in the United Kingdom—A retrospective cohort study
Source: Pharmacoepidemiol Drug Saf. 2022 Jun 10;31(8):892–900. doi: 10.1002/pds.5484 (PMC9542279; doi:10.1002/pds.5484)

**Supplementary figure 1: Period prevalence of antidepressant medication prescriptions in people diagnosed with type 2 diabetes**


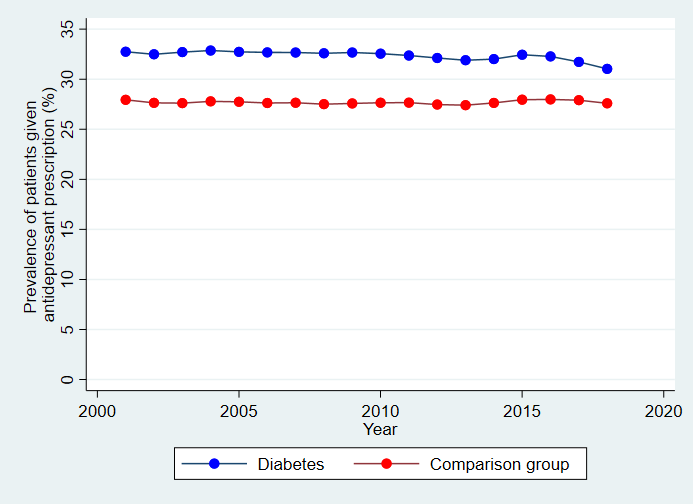


**Supplementary figure 2: Time to first prescription of anti-depressant medication in people with type 2 diabetes and the comparison group**


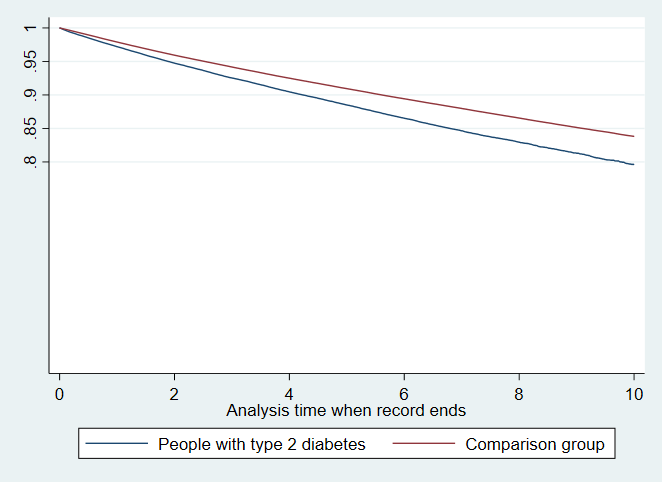

Supplement: Supplementary file 1 — Supplementary Figure S1 Period prevalence of antidepressant medication prescriptions in people diagnosed with type 2 diabetes Supplementary Figure S2: Time to first prescription of antidepressant medication in people with type 2 diabetes and the comparison group [file PDS-31-892-s002.docx]
