# Supplementary material for: Initiation of antidepressant medication in people with type 2 diabetes living in the United Kingdom—A retrospective cohort study
Source: Pharmacoepidemiol Drug Saf. 2022 Jun 10;31(8):892–900. doi: 10.1002/pds.5484 (PMC9542279; doi:10.1002/pds.5484)
Supplement: Supplementary file 2 — Appendix S1 Supporting Information [file PDS-31-892-s001.docx]

**Appendix 1: Read code lists**

**Diabetes**

medcode description

13AB.00 Diabetic lipid lowering diet

13AC.00 Diabetic weight reducing diet

13B1.00 Diabetic diet

1434.00 H/O: diabetes mellitus

14F4.00 H/O: Admission in last year for diabetes foot problem

1M8..00 Diabetic peripheral neuropathic pain

2BBF.00 Retinal abnormality - diabetes related

2BBJ.00 O/E - no right diabetic retinopathy

2BBK.00 O/E - no left diabetic retinopathy

2BBL.00 O/E - diabetic maculopathy present both eyes

2BBM.00 O/E - diabetic maculopathy absent both eyes

2BBP.00 O/E - right eye background diabetic retinopathy

2BBQ.00 O/E - left eye background diabetic retinopathy

2BBR.00 O/E - right eye preproliferative diabetic retinopathy

2BBS.00 O/E - left eye preproliferative diabetic retinopathy

2BBT.00 O/E - right eye proliferative diabetic retinopathy

2BBV.00 O/E - left eye proliferative diabetic retinopathy

2BBW.00 O/E - right eye diabetic maculopathy

2BBX.00 O/E - left eye diabetic maculopathy

2BBk.00 O/E - right eye stable treated prolif diabetic retino

2BBl.00 O/E - left eye stable treated prolif diabetic retinop

2BBo.00 O/E - sight threatening diabetic retinopathy

2BBr.00 Impair vision due diab retinop

2G51000 Foot abnormality - diabetes related

2G5A.00 O/E - Right diabetic foot at risk

2G5B.00 O/E - Left diabetic foot at risk

2G5C.00 Foot abnormality - diabetes related

2G5E.00 O/E - Right diabetic foot at low risk

2G5F.00 O/E - Right diabetic foot at moderate risk

2G5G.00 O/E - Right diabetic foot at high risk

2G5H.00 O/E - Right diabetic foot - ulcerated

2G5I.00 O/E - Left diabetic foot at low risk

2G5J.00 O/E - Left diabetic foot at moderate risk

2G5K.00 O/E - Left diabetic foot at high risk

2G5L.00 O/E - Left diabetic foot - ulcerated

2G5V.00 O/E - right chronic diabetic foot ulcer

2G5W.00 O/E - left chronic diabetic foot ulcer

2G5d.00 O/E - Left diabetic foot at increased risk

2G5e.00 O/E - Right diabetic foot at increased risk

3882.00 Diabetes well being questionnaire

3883.00 Diabetes treatment satisfaction questionnaire

42c1.00 HbA1 7 - 10% - borderline control

42c2.00 HbA1 > 10% - bad control

661M400 Diabetes self-management plan agreed

661N400 Diabetes self-management plan review

66A..00 Diabetic monitoring

66A1.00 Initial diabetic assessment

66A2.00 Follow-up diabetic assessment

66A3.00 Diabetic on diet only

66A4.00 Diabetic on oral treatment

66A5.00 Diabetic on insulin

66A8.00 Has seen dietician - diabetes

66A9.00 Understands diet - diabetes

66AA.11 Injection sites - diabetic

66AD.00 Fundoscopy - diabetic check

66AG.00 Diabetic drug side effects

66AH.00 Diabetic treatment changed

66AH000 Conversion to insulin

66AH100 Conversion to insulin in secondary care

66AH300 Conversion to non-insulin injectable medication

66AI.00 Diabetic - good control

66AJ.00 Diabetic - poor control

66AJ.11 Unstable diabetes

66AJ100 Brittle diabetes

66AJz00 Diabetic - poor control NOS

66AK.00 Diabetic - cooperative patient

66AL.00 Diabetic-uncooperative patient

66AM.00 Diabetic - follow-up default

66AN.00 Date diabetic treatment start

66AO.00 Date diabetic treatment stopp.

66AP.00 Diabetes: practice programme

66AQ.00 Diabetes: shared care programme

66AQ000 Unsuitable for diabetes year of care programme

66AQ100 Declined consent for diabetes year of care programme

66AR.00 Diabetes management plan given

66AS.00 Diabetic annual review

66AT.00 Annual diabetic blood test

66AU.00 Diabetes care by hospital only

66AV.00 Diabetic on insulin and oral treatment

66AW.00 Diabetic foot risk assessment

66AX.00 Diabetes: shared care in pregnancy - diabetol and obs

66AY.00 Diabetic diet - good compliance

66AZ.00 Diabetic monitoring NOS

66Aa.00 Diabetic diet - poor compliance

66Ab.00 Diabetic foot examination

66Ac.00 Diabetic peripheral neuropathy screening

66Ai.00 Diabetic 6 month review

66Ak.00 Diabetic monitoring - lower risk albumin excretion

66Al.00 Diabetic monitoring - higher risk albumin excretion

66Am.00 Insulin dose changed

66Ao.00 Diabetes type 2 review

66Ap.00 Insulin treatment initiated

66Aq.00 Diabetic foot screen

66Ar.00 Insulin treatment stopped

66As.00 Diabetic on subcutaneous treatment

66At.00 Diabetic dietary review

66At100 Type II diabetic dietary review

66At111 Type 2 diabetic dietary review

66Au.00 Diabetic erectile dysfunction review

66Av.00 Diabetic assessment of erectile dysfunction

66Az.00 High risk of diabetes mellitus annual review

66o5.00 Diabetic on oral treatment and glucagon-like peptide 1

66o6.00 Diabetic on insulin and glucagon-like peptide 1

6761.00 Diabetic pre-pregnancy counselling

679L000 Education in self management of diabetes

679R.00 Patient offered diabetes structured education program

67D8.00 Provision of diabetes clinical summary

67IJ100 Pre-conception advice for diabetes mellitus

68A7.00 Diabetic retinopathy screening

68A9.00 Diabetic retinopathy screening offered

68AB.00 Diabetic digital retinopathy screening offered

889A.00 Diab mellit insulin-glucose infus acute myocardial in

8A12.00 Diabetic crisis monitoring

8A13.00 Diabetic stabilisation

8B3l.00 Diabetes medication review

8BAi.00 Insulin passport completed

8BAj.00 Informed dissent not to carry insulin passport

8BAm.00 Insulin passport checked

8BAp.00 Insulin passport not checked

8BL2.00 Patient on maximal tolerated therapy for diabetes

8CA4100 Pt advised re diabetic diet

8CE0100 Insulin alert patient information booklet given

8CE0200 Insulin passport given

8CMW700 Diabetes clinical pathway

8CP2.00 Transition of diabetes care options discussed

8CR2.00 Diabetes clinical management plan

8CS0.00 Diabetes care plan agreed

8H2J.00 Admit diabetic emergency

8H3O.00 Non-urgent diabetic admission

8H4F.00 Referral to diabetologist

8H4e.00 Referral to diabetes special interest general practit

8H7C.00 Refer, diabetic liaison nurse

8H7f.00 Referral to diabetes nurse

8H7r.00 Refer to diabetic foot screener

8HBG.00 Diabetic retinopathy 12 month review

8HBH.00 Diabetic retinopathy 6 month review

8HHy.00 Referral to diabetic register

8HKE.00 Diabetology D.V. requested

8HLE.00 Diabetology D.V. done

8HME.00 Listed for Diabetology admissn

8HTE100 Referral to community diabetes clinic

8HTe.00 Referral to diabetes preconception counselling clinic

8HTi.00 Referral to multidisciplinary diabetic clinic

8HTk.00 Referral to diabetic eye clinic

8HVU.00 Private referral to diabetologist

8Hg4.00 Discharged from care of diabetes specialist nurse

8HgC.00 Discharged from diabetes shared care programme

8Hj1.00 Family/carer referral to diabetes structured education

8Hj4.00 Referral to DESMOND diabetes structured education programme

8Hl1.00 Referral for diabetic retinopathy screening

8Hl4.00 Referral to community diabetes specialist nurse

8Hlc.00 Referral to community diabetes service

8I3W.00 Diabetic foot examination declined

8I3X.00 Diabetic retinopathy screening refused

8I57.00 Patient held diabetic record declined

8I6F.00 Diabetic retinopathy screening not indicated

8I6G.00 Diabetic foot examination not indicated

8I83.00 Did not complete DESMOND diabetes structured educat program

8IAs.00 Diabetic dietary review declined

8IE2.00 Diabetes care plan declined

8IEQ.00 Referral to community diabetes specialist nurse decli

918T.00 Diabetes key contact

9360.00 Patient held diabetic record issued

93C4.00 Patient consent given for addition to diabetic regist

9M00.00 Informed consent for diabetes national audit

9M10.00 Informed dissent for diabetes national audit

9N0m.00 Seen in diabetic nurse consultant clinic

9N0n.00 Seen in community diabetes specialist clinic

9N0o.00 Seen in community diabetic specialist nurse clinic

9N1Q.00 Seen in diabetic clinic

9N1i.00 Seen in diabetic foot clinic

9N1o.00 Seen in multidisciplinary diabetic clinic

9N1v.00 Seen in diabetic eye clinic

9N2d.00 Seen by diabetologist

9N2i.00 Seen by diabetic liaison nurse

9N4I.00 DNA - Did not attend diabetic clinic

9N4p.00 Did not attend diabetic retinopathy clinic

9NJy.00 In-house diabetic foot screening

9NM0.00 Attending diabetes clinic

9NN8.00 Under care of diabetologist

9NN9.00 Under care of diabetes specialist nurse

9NN9000 Under care hos diab spec nurse

9NN9100 Under care com diab spec nurse

9NND.00 Under care of diabetic foot screener

9NiD.00 Did not attend DESMOND diabetes structured education program

9NiZ.00 Did not attend diabetes foot screening

9Nl4.00 Seen by general practitioner special interest in diab

9NlP100 Seen by diabetes speclst nurse

9OL..00 Diabetes monitoring admin.

9OL..11 Diabetes clinic administration

9OL1.00 Attends diabetes monitoring

9OL2.00 Refuses diabetes monitoring

9OL3.00 Diabetes monitoring default

9OL4.00 Diabetes monitoring 1st letter

9OL5.00 Diabetes monitoring 2nd letter

9OL6.00 Diabetes monitoring 3rd letter

9OL7.00 Diabetes monitor.verbal invite

9OL8.00 Diabetes monitor.phone invite

9OL9.00 Diabetes monitoring deleted

9OLA.00 Diabetes monitor. check done

9OLA.11 Diabetes monitored

9OLD.00 Diabetic patient unsuitable for digital retinal photo

9OLJ.00 DAFNE diabetes structured education programme complet

9OLK.00 DESMOND diabetes structured education programme completed

9OLN.00 Diabetes monitor invitation by SMS (short message ser

9OLZ.00 Diabetes monitoring admin.NOS

9Oy..00 Diabetes screening administration

9b92000 Diabetic medicine

9h4..00 Exception reporting: diabetes quality indicators

9h41.00 Excepted from diabetes qual indicators: Patient unsui

9h42.00 Excepted from diabetes quality indicators: Informed d

9h43.00 Excepted from diabetes qual indicators: service unava

9m0..00 Diabetic retinopathy screening administrative status

9m00.00 Eligible for diabetic retinopathy screening

9m01.00 Ineligible for diabetic retinopathy screening

9m02.00 Eligb temp inactv diab ret scr

9m03.00 Eligb perm inactv diab ret scr

9m04.00 Excluded from diabetic retinopathy screening

9m05.00 Excluded from diabetic retinopathy screening as moved

9m06.00 Excluded from diabetic retinopathy screening as decea

9m07.00 Excluded diabetc retinop screen as under care ophthal

9m08.00 Exclu diab ret screen as blind

9m0A.00 Declined diabetic retinop scrn

9m0B.00 Ex diab ret scr no cntct detls

9m0C.00 Excluded frm diabetic retinopathy screen as terminal

9m0D.00 Excluded from diabetic retinopthy screen as learn dis

9m0E.00 Excluded from diabetic retinopathy screen physical di

C10..00 Diabetes mellitus

C100.00 Diabetes mellitus with no mention of complication

C100000 Diabetes mellitus, juvenile type, no mention of compl

C100011 Insulin dependent diabetes mellitus

C100100 Diabetes mellitus, adult onset, no mention of complic

C100111 Maturity onset diabetes

C100112 Non-insulin dependent diabetes mellitus

C100z00 Diabetes mellitus NOS with no mention of complication

C101.00 Diabetes mellitus with ketoacidosis

C101000 Diabetes mellitus, juvenile type, with ketoacidosis

C101100 Diabetes mellitus, adult onset, with ketoacidosis

C101y00 Other specified diabetes mellitus with ketoacidosis

C101z00 Diabetes mellitus NOS with ketoacidosis

C102.00 Diabetes mellitus with hyperosmolar coma

C102100 Diabetes mellitus, adult onset, with hyperosmolar com

C102z00 Diabetes mellitus NOS with hyperosmolar coma

C103.00 Diabetes mellitus with ketoacidotic coma

C103100 Diabetes mellitus, adult onset, with ketoacidotic com

C103y00 Other specified diabetes mellitus with coma

C103z00 Diabetes mellitus NOS with ketoacidotic coma

C104.00 Diabetes mellitus with renal manifestation

C104.11 Diabetic nephropathy

C104100 Diabetes mellitus, adult onset, with renal manifestat

C104y00 Other specified diabetes mellitus with renal complica

C104z00 Diabetes mellitus with nephropathy NOS

C105.00 Diabetes mellitus with ophthalmic manifestation

C105000 Diabetes mellitus, juvenile type, + ophthalmic manife

C105100 Diabetes mellitus, adult onset, + ophthalmic manifest

C105y00 Other specified diabetes mellitus with ophthalmic com

C105z00 Diabetes mellitus NOS with ophthalmic manifestation

C106.00 Diabetes mellitus with neurological manifestation

C106.11 Diabetic amyotrophy

C106.12 Diabetes mellitus with neuropathy

C106.13 Diabetes mellitus with polyneuropathy

C106000 Diabetes mellitus, juvenile, + neurological manifesta

C106100 Diabetes mellitus, adult onset, + neurological manife

C106y00 Other specified diabetes mellitus with neurological c

C106z00 Diabetes mellitus NOS with neurological manifestation

C107.00 Diabetes mellitus with peripheral circulatory disorde

C107.11 Diabetes mellitus with gangrene

C107.12 Diabetes with gangrene

C107000 Diabetes mellitus, juvenile +peripheral circulatory d

C107100 Diabetes mellitus, adult, + peripheral circulatory di

C107200 Diabetes mellitus, adult with gangrene

C107400 NIDDM with peripheral circulatory disorder

C107y00 Other specified diabetes mellitus with periph circ co

C107z00 Diabetes mellitus NOS with peripheral circulatory dis

C109.00 Non-insulin dependent diabetes mellitus

C109.11 NIDDM - Non-insulin dependent diabetes mellitus

C109.12 Type 2 diabetes mellitus

C109.13 Type II diabetes mellitus

C109000 Non-insulin-dependent diabetes mellitus with renal co

C109011 Type II diabetes mellitus with renal complications

C109012 Type 2 diabetes mellitus with renal complications

C109100 Non-insulin-dependent diabetes mellitus with ophthalm

C109111 Type II diabetes mellitus with ophthalmic complicatio

C109112 Type 2 diabetes mellitus with ophthalmic complication

C109200 Non-insulin-dependent diabetes mellitus with neuro co

C109211 Type II diabetes mellitus with neurological complicat

C109212 Type 2 diabetes mellitus with neurological complicati

C109300 Non-insulin-dependent diabetes mellitus with multiple

C109311 Type II diabetes mellitus with multiple complications

C109312 Type 2 diabetes mellitus with multiple complications

C109400 Non-insulin dependent diabetes mellitus with ulcer

C109411 Type II diabetes mellitus with ulcer

C109412 Type 2 diabetes mellitus with ulcer

C109500 Non-insulin dependent diabetes mellitus with gangrene

C109511 Type II diabetes mellitus with gangrene

C109512 Type 2 diabetes mellitus with gangrene

C109600 Non-insulin-dependent diabetes mellitus with retinopa

C109611 Type II diabetes mellitus with retinopathy

C109612 Type 2 diabetes mellitus with retinopathy

C109700 Non-insulin dependent diabetes mellitus - poor contro

C109711 Type II diabetes mellitus - poor control

C109712 Type 2 diabetes mellitus - poor control

C109900 Non-insulin-dependent diabetes mellitus without compl

C109911 Type II diabetes mellitus without complication

C109912 Type 2 diabetes mellitus without complication

C109A00 Non-insulin dependent diabetes mellitus with mononeur

C109A11 Type II diabetes mellitus with mononeuropathy

C109A12 Type 2 diabetes mellitus with mononeuropathy

C109B00 Non-insulin dependent diabetes mellitus with polyneur

C109B11 Type II diabetes mellitus with polyneuropathy

C109B12 Type 2 diabetes mellitus with polyneuropathy

C109C00 Non-insulin dependent diabetes mellitus with nephropa

C109C11 Type II diabetes mellitus with nephropathy

C109C12 Type 2 diabetes mellitus with nephropathy

C109D00 Non-insulin dependent diabetes mellitus with hypoglyc

C109D11 Type II diabetes mellitus with hypoglycaemic coma

C109D12 Type 2 diabetes mellitus with hypoglycaemic coma

C109E00 Non-insulin depend diabetes mellitus with diabetic ca

C109E11 Type II diabetes mellitus with diabetic cataract

C109E12 Type 2 diabetes mellitus with diabetic cataract

C109F00 Non-insulin-dependent d m with peripheral angiopath

C109F11 Type II diabetes mellitus with peripheral angiopathy

C109F12 Type 2 diabetes mellitus with peripheral angiopathy

C109G00 Non-insulin dependent diabetes mellitus with arthropa

C109G11 Type II diabetes mellitus with arthropathy

C109G12 Type 2 diabetes mellitus with arthropathy

C109H00 Non-insulin dependent d m with neuropathic arthropath

C109H11 Type II diabetes mellitus with neuropathic arthropath

C109H12 Type 2 diabetes mellitus with neuropathic arthropathy

C109J00 Insulin treated Type 2 diabetes mellitus

C109J11 Insulin treated non-insulin dependent diabetes mellit

C109J12 Insulin treated Type II diabetes mellitus

C109K00 Hyperosmolar non-ketotic state in type 2 diabetes mel

C10A.00 Malnutrition-related diabetes mellitus

C10A000 Malnutrition-related diabetes mellitus with coma

C10A100 Malnutrition-related diabetes mellitus with ketoacido

C10A200 Malnutrition-related diabetes mellitus with renal com

C10A300 Malnutrit-related diabetes mellitus wth ophthalmic co

C10A400 Malnutrition-related diabetes mellitus wth neuro comp

C10A500 Malnutritn-relat diabetes melitus wth periph circul c

C10A600 Malnutrition-related diabetes mellitus with multiple

C10A700 Malnutrition-related diabetes mellitus without compli

C10AW00 Malnutrit-related diabetes mellitus with unspec compl

C10AX00 Malnutrit-relat diabetes mellitus with other spec com

C10B.00 Diabetes mellitus induced by steroids

C10B000 Steroid induced diabetes mellitus without complicatio

C10C.00 Diabetes mellitus autosomal dominant

C10D.00 Diabetes mellitus autosomal dominant type 2

C10D.11 Maturity onset diabetes in youth type 2

C10F.00 Type 2 diabetes mellitus

C10F.11 Type II diabetes mellitus

C10F000 Type 2 diabetes mellitus with renal complications

C10F011 Type II diabetes mellitus with renal complications

C10F100 Type 2 diabetes mellitus with ophthalmic complication

C10F111 Type II diabetes mellitus with ophthalmic complicatio

C10F200 Type 2 diabetes mellitus with neurological complicati

C10F211 Type II diabetes mellitus with neurological complicat

C10F300 Type 2 diabetes mellitus with multiple complications

C10F311 Type II diabetes mellitus with multiple complications

C10F400 Type 2 diabetes mellitus with ulcer

C10F411 Type II diabetes mellitus with ulcer

C10F500 Type 2 diabetes mellitus with gangrene

C10F511 Type II diabetes mellitus with gangrene

C10F600 Type 2 diabetes mellitus with retinopathy

C10F611 Type II diabetes mellitus with retinopathy

C10F700 Type 2 diabetes mellitus - poor control

C10F711 Type II diabetes mellitus - poor control

C10F900 Type 2 diabetes mellitus without complication

C10F911 Type II diabetes mellitus without complication

C10FA00 Type 2 diabetes mellitus with mononeuropathy

C10FA11 Type II diabetes mellitus with mononeuropathy

C10FB00 Type 2 diabetes mellitus with polyneuropathy

C10FB11 Type II diabetes mellitus with polyneuropathy

C10FC00 Type 2 diabetes mellitus with nephropathy

C10FC11 Type II diabetes mellitus with nephropathy

C10FD00 Type 2 diabetes mellitus with hypoglycaemic coma

C10FD11 Type II diabetes mellitus with hypoglycaemic coma

C10FE00 Type 2 diabetes mellitus with diabetic cataract

C10FE11 Type II diabetes mellitus with diabetic cataract

C10FF00 Type 2 diabetes mellitus with peripheral angiopathy

C10FF11 Type II diabetes mellitus with peripheral angiopathy

C10FG00 Type 2 diabetes mellitus with arthropathy

C10FG11 Type II diabetes mellitus with arthropathy

C10FH00 Type 2 diabetes mellitus with neuropathic arthropathy

C10FH11 Type II diabetes mellitus with neuropathic arthropath

C10FJ00 Insulin treated Type 2 diabetes mellitus

C10FJ11 Insulin treated Type II diabetes mellitus

C10FK00 Hyperosmolar non-ketotic state in type 2 diabetes mel

C10FK11 Hyperosmolar non-ketotic state in type II diabetes me

C10FL00 Type 2 diabetes mellitus with persistent proteinuria

C10FL11 Type II diabetes mellitus with persistent proteinuria

C10FM00 Type 2 diabetes mellitus with persistent microalbumin

C10FM11 Type II diabetes mellitus with persistent microalbumi

C10FN00 Type 2 diabetes mellitus with ketoacidosis

C10FN11 Type II diabetes mellitus with ketoacidosis

C10FP00 Type 2 diabetes mellitus with ketoacidotic coma

C10FP11 Type II diabetes mellitus with ketoacidotic coma

C10FQ00 Type 2 diabetes mellitus with exudative maculopathy

C10FQ11 Type II diabetes mellitus with exudative maculopathy

C10FR00 Type 2 diabetes mellitus with gastroparesis

C10FR11 Type II diabetes mellitus with gastroparesis

C10FS00 Maternally inherited diabetes mellitus

C10G.00 Secondary pancreatic diabetes mellitus

C10G000 Secondary pancreatic diabetes mellitus without compli

C10H.00 Diabetes mellitus induced by non-steroid drugs

C10H000 DM induced by non-steroid drugs without complication

C10J.00 Insulin autoimmune syndrome

C10J000 Insulin autoimmune syndrome without complication

C10K.00 Type A insulin resistance

C10K000 Type A insulin resistance without complication

C10M.00 Lipoatrophic diabetes mellitus

C10M000 Lipoatrophic diabetes mellitus without complication

C10N.00 Secondary diabetes mellitus

C10N000 Secondary diabetes mellitus without complication

C10N100 Cystic fibrosis related diabetes mellitus

C10y.00 Diabetes mellitus with other specified manifestation

C10y000 Diabetes mellitus, juvenile, + other specified manife

C10y100 Diabetes mellitus, adult, + other specified manifesta

C10yy00 Other specified diabetes mellitus with other spec com

C10yz00 Diabetes mellitus NOS with other specified manifestat

C10z.00 Diabetes mellitus with unspecified complication

C10z000 Diabetes mellitus, juvenile type, + unspecified compl

C10z100 Diabetes mellitus, adult onset, + unspecified complic

C10zy00 Other specified diabetes mellitus with unspecified co

C10zz00 Diabetes mellitus NOS with unspecified complication

Cyu2.00 [X]Diabetes mellitus

Cyu2000 [X]Other specified diabetes mellitus

Cyu2100 [X]Malnutrit-relat diabetes mellitus with other spec

Cyu2200 [X]Malnutrit-related diabetes mellitus with unspec co

Cyu2300 [X]Unspecified diabetes mellitus with renal complicat

F171100 Autonomic neuropathy due to diabetes

F35z000 Diabetic mononeuritis NOS

F372.00 Polyneuropathy in diabetes

F372.11 Diabetic polyneuropathy

F372.12 Diabetic neuropathy

F372000 Acute painful diabetic neuropathy

F372100 Chronic painful diabetic neuropathy

F372200 Asymptomatic diabetic neuropathy

F381300 Myasthenic syndrome due to diabetic amyotrophy

F381311 Diabetic amyotrophy

F3y0.00 Diabetic mononeuropathy

F420.00 Diabetic retinopathy

F420000 Background diabetic retinopathy

F420100 Proliferative diabetic retinopathy

F420200 Preproliferative diabetic retinopathy

F420300 Advanced diabetic maculopathy

F420400 Diabetic maculopathy

F420500 Advanced diabetic retinal disease

F420600 Non proliferative diabetic retinopathy

F420700 High risk proliferative diabetic retinopathy

F420800 High risk non proliferative diabetic retinopathy

F420z00 Diabetic retinopathy NOS

F440700 Diabetic iritis

F464000 Diabetic cataract

G73y000 Diabetic peripheral angiopathy

K01x100 Nephrotic syndrome in diabetes mellitus

K08yA00 Proteinuric diabetic nephropathy

K08yA11 Clinical diabetic nephropathy

K27y700 Erectile dysfunction due to diabetes mellitus

Kyu0300 [X]Glomerular disorders in diabetes mellitus

L180600 Pre-existing diabetes mellitus, non-insulin-dependent

L180700 Pre-existing malnutrition-related diabetes mellitus

L180X00 Pre-existing diabetes mellitus, unspecified

Lyu2900 [X]Pre-existing diabetes mellitus, unspecified

M037200 Cellulitis in diabetic foot

M271000 Ischaemic ulcer diabetic foot

M271100 Neuropathic diabetic ulcer - foot

M271200 Mixed diabetic ulcer - foot

N030000 Diabetic cheiroarthropathy

N030011 Diabetic cheiropathy

N030100 Diabetic Charcot arthropathy

Q441.00 Neonatal diabetes mellitus

R054200 [D]Gangrene of toe in diabetic

R054300 [D]Widespread diabetic foot gangrene

SL23z00 Insulins or antidiabetic poisoning NOS

TJ23.00 Adverse reaction to insulins and antidiabetic agents

TJ23z00 Adverse reaction to insulins and antidiabetic agents

U602311 [X] Adverse reaction to insulins and antidiabetic age

U60231E [X] Adverse reaction to insulins and antidiabetic age

ZC2C800 Dietary advice for diabetes mellitus

ZC2C911 Diet advice for insulin-dependent diabetes

ZC2CA00 Dietary advice for type II diabetes

ZC2CA11 Dietary advice non-insulin-dependent diabetes

ZL22500 Under care of diabetic liaison nurse

ZL62500 Referral to diabetes nurse

ZL62600 Referral to diabetic liaison nurse

ZLA2500 Seen by diabetic liaison nurse

ZLD7500 Discharge by diabetic liaison nurse

ZRB4.00 Diabetes clinic satisfaction questionnaire

ZRB4.11 CSQ - Diabetes clinic satisfaction questionnaire

ZRB5.00 Diabetes treatment satisfaction questionnaire

ZRB5.11 DTSQ - Diabetes treatment satisfaction questionnaire

ZRB6.00 Diabetes wellbeing questionnaire

ZRB6.11 DWBQ - Diabetes wellbeing questionnaire

ZRbH.00 Perceived control of insulin-dependent diabetes

ZV65312 [V]Dietary counselling in diabetes mellitus

ZV6DA00 [V]Admitted for commencement of insulin

ZV6DB00 [V]Admitted for conversion to insulin

ahdcode

1009100000 diabetes annual check

1009111000 diabetes current status

1009120000 diabetes insulin dosage

1001400140 hb a1c - diabetic control

1001400327 diabetic retinopathy screening
